# A Comparative Study of Two Blast-Induced Traumatic Brain Injury Models: Changes in Monoamine and Galanin Systems Following Single and Repeated Exposure

**DOI:** 10.3389/fneur.2018.00479

**Published:** 2018-06-20

**Authors:** Lizan Kawa, Alaa Kamnaksh, Joseph B. Long, Ulf P. Arborelius, Tomas Hökfelt, Denes V. Agoston, Mårten Risling

**Affiliations:** ^1^Department of Neuroscience, Karolinska Institutet, Stockholm, Sweden; ^2^Department of Anatomy, Physiology and Genetics, Uniformed Services, University, Bethesda, MD, United States; ^3^Blast-Induced Neurotrauma Branch, Center for Military Psychiatry and Neuroscience, Walter Reed Army Institute of Research, Silver Spring, MD, United States

**Keywords:** anxiety, catecholamines, dorsal raphe nucleus, locus coeruleus, animal models, neuropeptide, post-traumatic stress disorder, transmitter coexistence

## Abstract

Repeated mild blast-induced traumatic brain injury (rmbTBI), caused by recurrent exposure to low levels of explosive blast, is a significant concern for military health systems. However, the pathobiology of rmbTBI is currently poorly understood. Animal models are important tools to identify the molecular changes of rmbTBI, but comparisons across different models can present their own challenges. In this study, we compared two well-established rodent models of mbTBI, the “KI model” and the “USU/WRAIR model.” These two models create different pulse forms, in terms of peak pressure and duration. Following single and double exposures to mild levels of blast, we used *in situ* hybridization (ISH) to assess changes in mRNA levels of tyrosine hydroxylase (TH), tryptophan hydroxylase (TPH2), and galanin in the locus coeruleus (LC) and dorsal raphe nucleus (DRN). These systems and their transmitters are known to mediate responses to stress and anxiety. We found increased mRNA levels of TH, TPH2 and galanin in the LC and DRN of single-exposed rats relative to sham rats in the KI but not the USU/WRAIR model. Sham mRNA values measured in the USU/WRAIR model were substantially higher than their KI counterparts. Double exposure caused similarly significant increases in mRNA values in the KI model but not the USU/WRAIR model, except TPH2 and galanin levels in the DRN. We detected no cumulative effect of injury in either model at the used inter-injury interval (30 min), and there were no detectable neuropathological changes in any experimental group at 1 day post-injury. The apparent lack of early response to injury as compared to sham in the USU/WRAIR model is likely caused by stressors (e.g., transportation and noise), associated with the experimental execution, that were absent in the KI model. This study is the first to directly compare two established rodent models of rmbTBI, and to highlight the challenges of comparing findings from different animal models. Additional studies are needed to understand the role of stress, dissect the effects of psychological and physical injuries and to identify the window of increased cerebral vulnerability, i.e., the inter-injury interval that results in a cumulative effect following repeated blast exposure.

## Introduction

Exposure to explosive blast causes a specific form of traumatic brain injury (TBI), termed blast-induced TBI or bTBI (1). The most frequent form of bTBI is mild; due to the mild and transient nature of clinical symptoms that follow. Soldiers are frequently exposed to a second, third, or more mild blasts (2). Exposure to additional blasts within the window of increased cerebral vulnerability, i.e., when the brain is still recovering from the initial impact, can have a cumulative effect that results in more severe acute symptoms and long-term pathological implications (3). Post-concussive symptoms (PCS) following mbTBI, and the possibility that symptoms are compounded by repeated exposures, have been extensively reported in the literature (4–7). Symptom onset can be immediate but also transient, or develop over time and become chronic (4, 5). PCS can include cognitive impairments, such as problems with memory or concentration, visual disturbances, tinnitus and headaches, as well as mood disorders, such as anxiety or depression, increased irritability or rage (8, 9). Some of these symptoms are also observed in post-traumatic stress disorder (PTSD) that can be caused by exposure(s) to psychological stress without evidence of physical injury (9). The exact pathobiology of PTSD is currently poorly understood, but alterations in the catecholamine, serotonin, and galanin systems have been implicated in the neurobehavioral abnormalities (10).

Animal models have played a fundamental role in the identification of disease mechanisms, diagnostic and molecular targets, and in testing therapeutic approaches. Animal models have also played a key role in recreating the physical and biological components of bTBI under controlled, reproducible conditions (11, 12). Apart from using free-field blast exposures with explosives, there are two basic models for generating blast overpressure that can be employed within the confinements of laboratory conditions. One is based on the design by Clemedson, which uses low quantities of explosives in a modified artillery piece (or blast tube) to generate a blast wave (13, 14). We refer to this model, used at the Karolinska Institutet, as the “KI model.” The other, more widely used model utilizes compressed air (or another driver gas, such as Helium) to generate a shock wave through the sudden burst of a membrane separating the compression and expansion chambers of the shock tube (15). This model, first described by Elsayed (16) and used at the Uniformed Services University (USU) and Walter Reed Army Institute of Research (WRAIR), is herein referred to as the “USU/WRAIR” model.

We have previously reported that exposing rats to a single mild blast in the KI model results in changes in the classical noradrenergic and serotonergic neurotransmitter systems in various forebrain regions, and of their rate-limiting enzymes in the lower brainstem (17). In a separate study, we also presented changes in the transcripts of the neuropeptide galanin and its receptors following exposure to a single mild blast (18). We consider these findings important, because they provide the rationale for developing potential pharmacological treatments to mitigate PCS; however, our findings need to be independently replicated. In addition, how these transmitter systems respond to repeated mild blasts remains to be examined.

Using the USU/WRAIR model, we have previously found that single exposure to mild blast overpressure triggers neurobehavioral changes, including increased anxiety, impaired learning and memory (19–21). At the molecular level, we identified metabolic and neuroinflammatory changes, as well as axonal, neuronal, and glial damage in behaviorally-relevant brain regions and in serum. We also found that repeated exposure to mild blast overpressure has a cumulative effect on the brain as indicated by both proteomics and diffusion tensor imaging (DTI) (22). Again, these findings need to be independently verified using additional outcome measures for bTBI.

An important consideration is that the majority of TBI studies are carried out using different blast models, strains and ages of rodents, to investigate various outcome measures at a range of terminal time points. Furthermore, where there are repeated exposures, the latency between injuries ranges from minutes to days between studies. Therefore, bTBI studies have produced results that are as heterogeneous as the disease itself. This paper is a step toward increasing translatability between experimental bTBI models and increasing confidence in the field's findings by replicating, combining, and building on previous findings. In line with this aim, we employed two well-established models of bTBI—the KI blast tube vs. USU/WRAIR shock tube—in two different laboratories using identical rat strains, ages, and anesthesia to compare the effects of single and repeated mild blast exposure on the transcript levels of the rate-limiting enzymes tyrosine hydroxylase (TH) and tryptophan hydroxylase 2 (TPH2), and of galanin in the lower brainstem using *in situ* hybridization (ISH). We also used immunohistochemistry to look for degenerating neurons and signs of axonal injury in the hippocampus.

## Materials and methods

### Experimental groups and manipulations

This study is composed of two separate experiments, one carried out at the Karolinska Institutet in Stockholm, Sweden (KI model) and the other one at the Uniformed Services University and Walter Reed Army Institute of Research (USU/WRAIR model), respectively, in Bethesda and Silver Spring, Maryland, USA. At KI, 22 male Sprague Dawley rats (Taconic, Ry, Denmark), weighing 250–320 g, were used. Animals were handled in accordance with the Swedish National Guidelines for Animal Experiments, and approved by the Stockholm Animal Care and Use Ethics Committee (Stockholm Norra Djurförsöksetiska Nämnd). Here the animals were separated into 4 groups: single exposed (*n* = 5), single sham (*n* = 5), double exposed (*n* = 6), and double sham (*n* = 6).

At USU, 16 male Sprague Dawley rats (Charles River Laboratories, Wilmington, MA), weighing 250–320 g, were used. Animals were housed in pairs in standard rat cages with built-in filters and food and water *ad libitum*. All experiments were performed according to protocol approved by the Institutional Animal Care and Use Committee at USU. The rats were divided into 4 groups: single exposed (*n* = 4), single sham (*n* = 4), double exposed (*n* = 4), and double sham (*n* = 4).

Animal handling and treatments were conducted in compliance with the Animal Welfare Act and other Federal statutes and regulations related to animals and experiments involving animals, and adhered to principles stated in the Guide to the Care and Use of Laboratory Animals, National Research Council. The facilities are fully accredited by the Association for Assessment and Accreditation of Laboratory Animal Care International.

### Anesthesia

At both sites, rats were anesthetized in an identical fashion using induction chambers with a 4% isoflurane-air mixture (*KI*: Janssen, Stockholm, Sweden; *WRAIR*: Forane; Baxter Healthcare Corporation, Deerfield, IL, USA). Sham animals were anesthetized for 6 min for the 1st exposure, and 3 min for the 2nd exposure. Blast-exposed groups underwent the same anesthesia procedure with the addition of 1 or 2 blast overpressure exposures. The interval between inductions/exposures was ~30 min.

### KI model

Animals used in the KI experiments were stored at the same facility. All animal handling, experiments, and sacrifice were carried out in the same laboratory; no transportation or other stressors were included.

A validated blast tube, designed by Clemedson, was used for the exposures (13, 14). Five grams of Swedish Army plastic explosive containing m/46, 86% pentaerythritol tetranitrate (PETN), and mineral oil was used with a NONEL ignition (Nobel, Sweden). Anesthetized animals were placed in a rigid metallic holder that protects all parts of the body, except the head, and prevents acceleration movements of the head relative to the rest of the body. The head is supported from below and on the side that is not facing the detonation. This support reduces acceleration movements as revealed by high speed video (30,000 frames/s, unpublished data of Dr. Johan Davidsson). We can see the first sign of a pressure wave induced movement after 1 ms. The head hits the support behind the head at around 3 ms and returns to the original position at 6.6 ms. After around 9 ms there is a second hit to the support. These movements are clearly below the thresholds previously reported for axonal injury in our model of rotational TBI (23).

**Figure 1:**
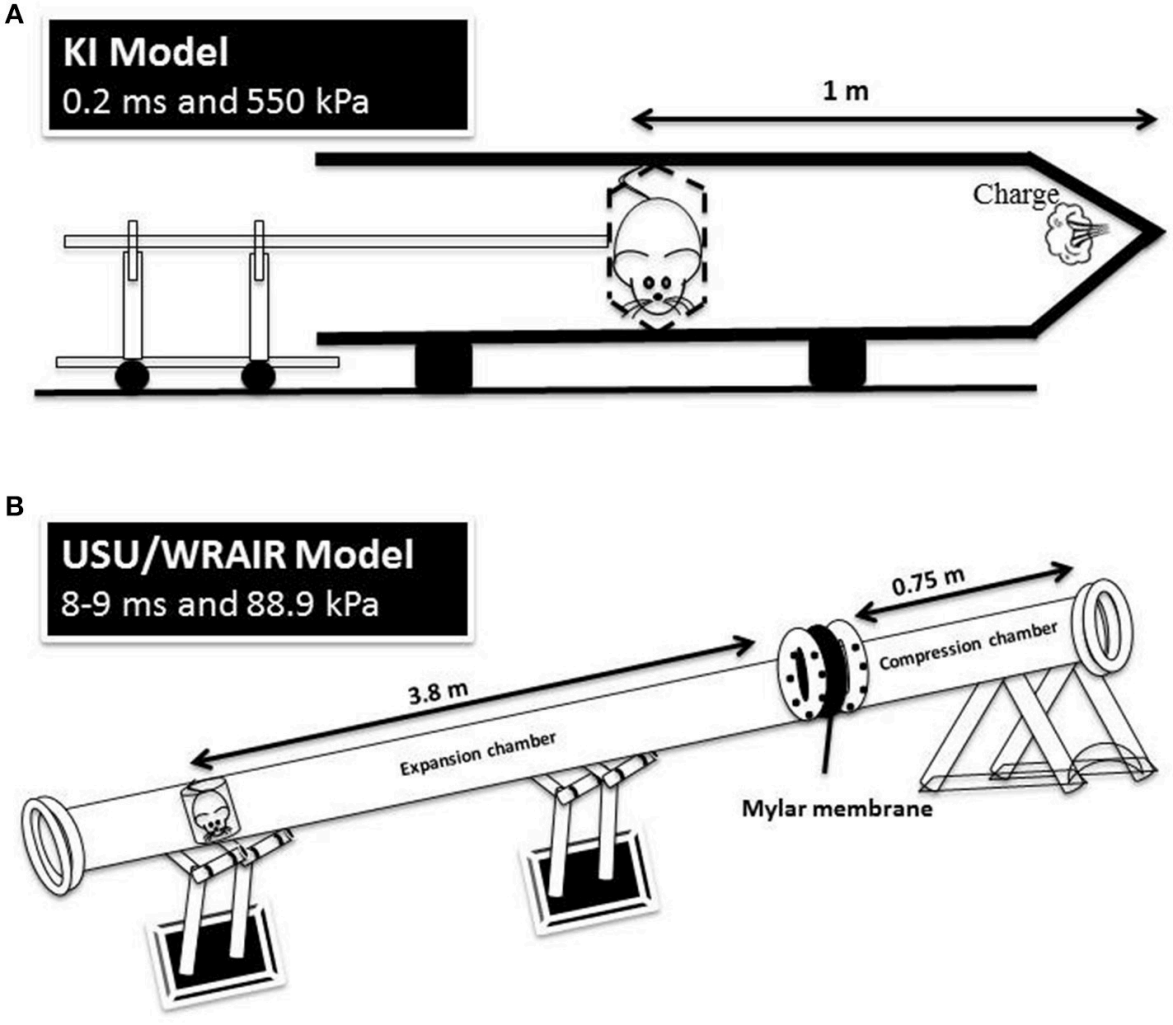
A schematic representation of the KI model **(A)** and USU/WRAIR model **(B)**. The positive phase durations and peak pressures are measured at the animal level in both models.

The holder was subsequently mounted into the 1.5 m metal tube, in a transverse prone position, at a distance of 1 m from the charge, with the rat's right side facing the blast source. Five grams of the explosive were then detonated, triggering a simple Friedlander-type blast wave at the surface of the animal with a peak pressure of 800 kPa and positive phase duration of 0.25 ms. A schematic of the KI model is depicted in Figure 1.

### USU/WRAIR model

On the day of the exposures the animals, housed at USU, were loaded into a cargo van and transported to WRAIR, where the exposures were conducted. The trip on common roads is ~8 miles long and takes about 20 min one way. Upon arrival at WRAIR, animal cages were unloaded and moved to the blast facility. For the duration of the exposures, cages were stored in the preparatory area of the blast room, where there was human traffic and various acoustic disturbances, including multiple rounds of blasting.

The shock tube is a 5.25 m long, 0.3 m in diameter, horizontally mounted circular steel tube. It is divided into a 0.75 m compression chamber and a 4.5 m expansion chamber by Mylar® sheets (Du Pont Co, Wilmington, DE, USA) (15, 16). Total pressure generated in the shock tube is dependent on the thickness of the Mylar membranes used. For the present exposures, Mylar membrane gauge thickness was 1400, producing an average peak pressure of 88.9 kPa and a positive phase duration of 8–9 ms (Figure 1, Table 1). Anesthetized rats wearing body protection were placed in the shock tube's metallic holder in a transverse prone position, at a distance of 3.8 m from the membrane with the right side of the animal facing the incident blast overpressure. At the completion of the exposures, animals were transported back to USU.

**Table 1:** Pressure recordings of the WRAIR model.

**Measured from pencil probe**				
Mylar membrane thickness	750	1,000	1,400 milliinches	Determination
Rupture pressure	32.33	43.25	59.38 psi	Measured
Reflected pressure	26.9	32.8	40.3 psi	Measured
Total pressure (static+ dynamic)	13.2	16.5	20 psi	Measured
Static pressure	8	9.5	11 psi	Measured
Dynamic pressure	5.2	7	9 psi	Calculated
Rise time	12.5	11.25	11.25 μs	Calculated from pitot guage
Positive phase duration (static)	6.34	7.4	8.5 ms	Calculated
Impulse (static)	226	309	388 kPa*ms	Calculated
Shock velocity	1.3	1.34	1.42 M	Calculated
Wind velocity	151.8	161.81	204.72 m/s	Calculated from R-H equation

### Tissue collection

One day after the injuries, animals were deeply anesthetized at both sites using isoflurane inhalant until a tail pinch produced no reflex movement. Animals were then decapitated, the brains removed, instantly fresh frozen, and stored at −80°C until further processing.

WRAIR samples were sent to KI for *in situ* hybridization (ISH) and immunohistochemistry (IHC) analysis, taking the appropriate precautions to maintain the quality of the samples.

### *In situ* hybridization

All samples for ISH were processed together in Stockholm, as described previously in detail (17). Briefly, serial 14 μm thick coronal sections were cut at the level of the locus coeruleus (LC) and dorsal raphe nucleus (DRN), using Cryo-Star HM 560 M (MICROM International GmbH, Heidelberg, Germany). Oligonucleotides complementary to rat mRNA for galanin, TH, and TPH2 (Table 2) were labeled with deoxyadenosine 5′triphosphate α-P^33^ (Perkin Elmer, Boston, MA, USA) at the 3′-end using terminal deoxynucleotidyltransferase (Thermo Scientific, Waltham, MA, USA). Sections were air-dried and incubated with the oligonucleotide probe and, post-hybridization, sections were rinsed, air-dried and dipped in liquid photo emulsion NTB2 (Kodak, Rochester, NY, USA). Slides were developed using D19 developer (Kodak), followed by AL-4 fixative (Kodak) and mounting in glycerol-phosphate. Dark field photomicrographs were captured in a Nikon Eclipse E-600 microscope connected to a digital camera (Digital Sight, U1; Nikon). The images were analyzed according to the mean gray density (MGD) of the mRNA signal in the regions of interest using ImageJ 1.48 (NIH).

**Table 2:** Probes used for ISH.

**Probe**	**Gene Bank accession no**.	**Primers**
**PRIMERS USED FOR OLIGO** ***IN SITU*** **HYBRIDIZATION**		
Galanin	NM_017139	GGTGCACAGTGGGTGTGGTCTCAGGACTGCTCT
		ATGCCAGGCAGGCTGTCGAGGGCCCCGGCCTCT
		GTGCGGACGATATTGCTCTCAGGCAGGGGTACA
		CCCGAGCCCCAGAGTGGCTGACAGGGTTGCAACCAACAGGAGCCAGGC
		TTGTCAATGGCATGTGGGCCCAGAAGGTAGCCA
TH	NM_012740	GCG CTG GAT ACG AGA GGC ATA GTT CCT GAG CTT GTC
TPH2	NM_017139	TCC TCC GTC CAA ATG TTG TCA GGT GGA TTC AGC GTC ACA ATG GTG GTC

#### Immunohistochemistry

Cryostat sections (see above) from the hippocampus were used to stain for degenerating neurons using Fluoro Jade B (FJ-B) and for β-amyloid precursor protein (APP) accumulation. *FJ staining*: sections were air dried, fixed in 4% formaldehyde, and rinsed in phosphate-buffered saline (PBS). The slides were subsequently dipped in dH_2_O, followed by potassium permanganate (KMnO_4_), and rinsed with dH_2_O. Slides were then soaked in FJ solution (Fluoro Jade B, Merk Millipore AG310, Darmstadt, Germany), washed in dH_2_O, and placed on a hot plate, dipped in xylene and mounted with Entellan (Merck, Darmstadt, Germany).

#### APP staining

The sections were fixed in ice cold methanol, shortly dipped in ice cold acetone, rinsed in PBS, and incubated in a humid chamber overnight with a solution of 0.3% Triton, 5% bovine serum albumin, and 0.1% sodium azide in 0.01M PBS and a rabbit poly-clonal antibody against APP (dilution 1:400; Life Technologies, 51-2700; Stockholm, Sweden). The following day sections were rinsed in PBS and incubated for 1 h with 0.01 M PBS, 0.1% sodium azide, 0.3% Triton, and processed with an Alexa Fluor 488 conjugated anti-rabbit IgG (dilution 1:400; Jackson ImmunoResearch, Suffolk, UK). Sections were rinsed and mounted in a mixture of glycerol and PBS (1:3), and then cover-slipped. Slides were viewed in a microscope equipped with epifluorescence (Eclipse E600, Nikon, Tokyo, Japan).

### Statistical analyses

GraphPad Prism version 6 (GraphPad Software, CA) was used to perform all statistical analyses. Signal intensity data were analyzed using one-way analysis of variance (ANOVA) followed by Tukey-Kramer Multiple Comparison Test. All data are presented as the mean ± standard error, where statistically significant data are highlighted: ^*^p < 0.05, ^**^p < 0.01, ^***^p < 0.001, ^****^p < 0.0001. All transcript levels were normalized to KI single sham levels for graphical purposes.

## Results

### *In situ* hybridization

**Figure 2:**
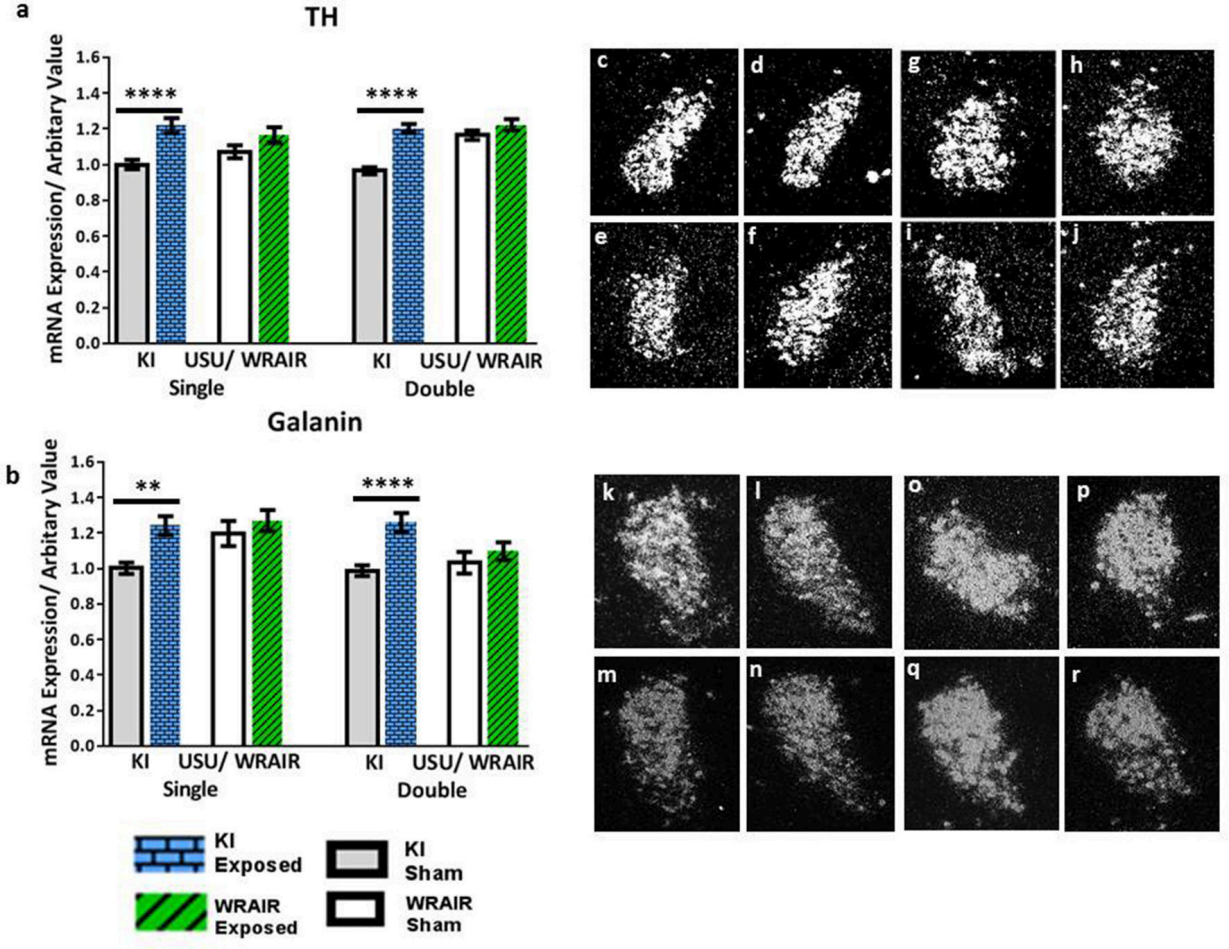
ISH analysis of transcript levels for TH and galanin in the LC following exposure to single or double mbTBI, using two different models of TBI, in two different laboratories. Quantification of transcript levels of TH **(a)** and galanin **(b)** revealed that both were significantly increased bilaterally at 1 day post-exposure in the single and double exposed groups, relative to their respective shams using the KI model. While the same trend was seen in exposed groups vs. shams in the USU/WRAIR model, the elevations were not statistically significant in any of the transcripts. There did not appear to be a cumulative effect of repeated exposure in either model. **(c–r)** Representative dark field ISH photomicrographs of emulsion-dipped sections show the distribution and levels of TH **(c–j)** and galanin **(k–r)** transcripts levels. TH mRNA levels in the single **(c)**, and double **(d)** exposed groups in the LC, relative to sham single **(e)**, and double **(f)** groups using the KI model. **(g–j)** Show TH mRNA distribution and levels in the single **(g)**, and double **(h)** exposed groups using the USU/WRAIR mbTBI model, and their respective sham groups; single **(i)**, and double **(j)**. Photomicrographs **(k–r)** show galanin transcript levels: the single **(k)** and double **(l)** exposed groups using the KI model, and their respective shams, single **(m)** and double **(n)**; the single **(o)**, and double exposed **(p)**, and single **(q)**, and double **(r)** sham groups, using the USU/WRAIR model. Data are presented as mean ± SEM (^**^p < 0.01, ^****^p < 0.0001). ISH, *in situ* hybridization; LC, locus coeruleus; TH, tyrosine hydroxylase.

In the LC, transcript levels of the biosynthetic enzyme TH and the neuropeptide galanin showed a significant, bilateral upregulation at 1 d post-exposure in the KI model (Figure 2). Analysis of the ISH images revealed that the increases in TH mRNA levels were statistically significant in both the single and double exposed groups relative to their sham groups (*p* < 0.0001; Figure 2a). Similarly, the elevations in galanin mRNA levels were also statistically significant in both the single and double exposed group in the KI model; however, the increase was more pronounced after double exposure (*p* < 0.0001; Figure 2b) compared to a single blast (*p* < 0.01; Figure 2b). In contrast, we only observed an upward trend in TH and galanin transcript levels in the WRAIR model's injured groups compared to their respective sham groups, but statistical significance was not reached (Figures 2a,b).

**Figure 3:**
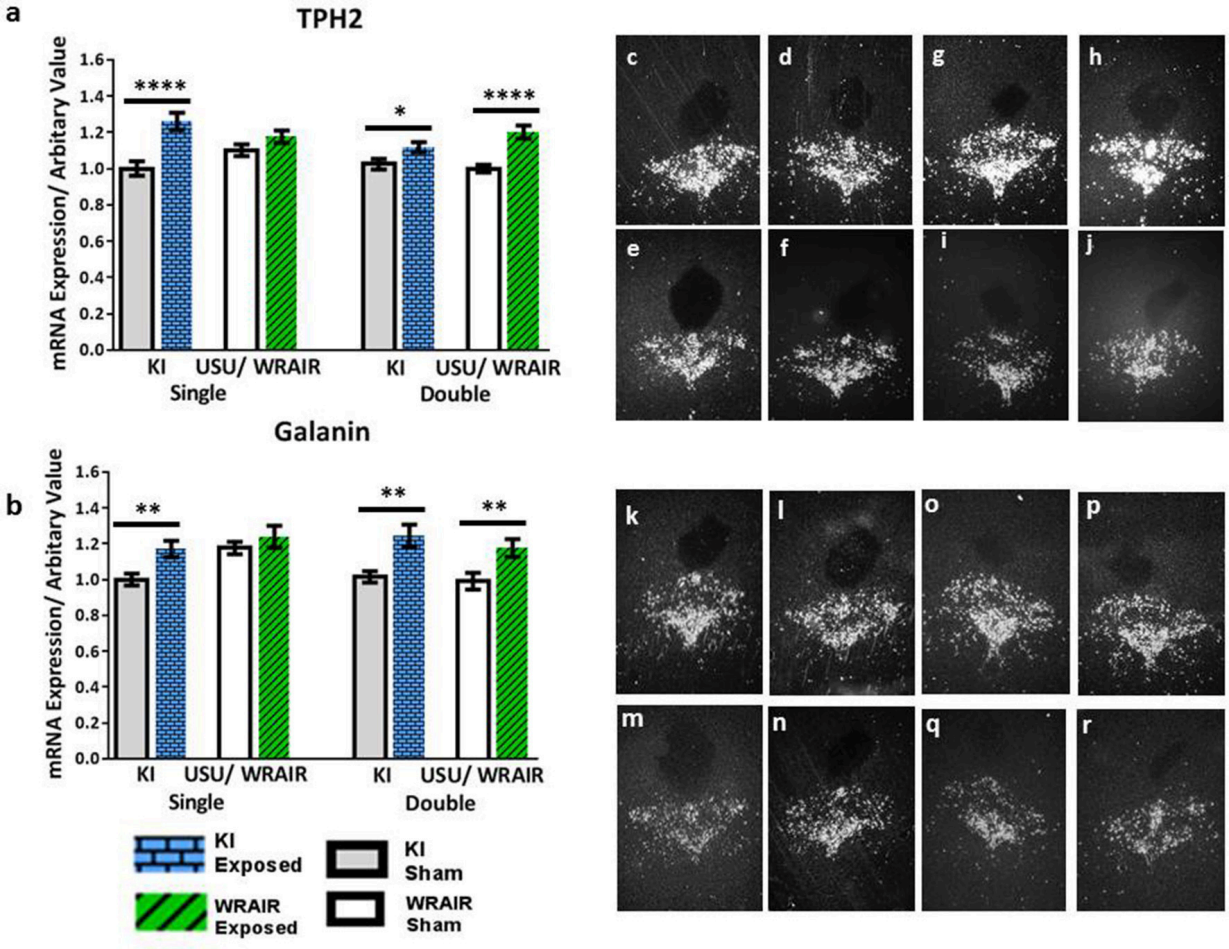
ISH analysis of transcript levels for TPH2 and galanin in the mid/caudal DRN following exposure to single or double mbTBI, using two different models of TBI, in two different laboratories. Quantification of transcript levels of TPH2 **(a)** and galanin **(b)** mRNA levels showed a significant increase at 1 day post-exposure in the single and double exposed groups relative to their respective shams using the KI model. While the same trend of an elevation was also observed in exposed groups vs. shams using the USU/WRAIR model, this was only statistically significant in the double exposure group. There did not appear to be a cumulative effect of repeated exposure in either model. **(c–r)** Representative dark field ISH photomicrographs of emulsion-dipped sections show distribution and levels of TPH2 **(c–j)** and galanin **(k–r)** transcripts. TPH2 mRNA levels in the single **(c)**, and double **(d)** exposed groups, relative to sham single **(e)**, and double **(f)** groups using the KI model. **(g–j)** Show single **(g)**, and double **(h)** exposed groups using the USU/WRAIR model, and their respective single **(i)**, and double **(j)** sham groups. Photomicrographs **(k-r)** show galanin transcript levels: the single **(k)** and double **(l)** exposed groups using the KI model, and their respective shams, single **(m)** and double **(n)**; the single **(o)**, and double exposed **(p)**, and single **(q)**, and double **(r)** sham groups, using the USU/WRAIR model. Data are presented as mean ± SEM (^*^p < 0.05, ^**^p < 0.01, ^****^p < 0.0001). ISH, *in situ* hybridization; DRN, dorsal raphe nucleus; TPH2, tryptophan hydroxylase 2.

In the DRN of rats processed for the KI model, an increase in the transcript levels of TPH2 and galanin was also observed in the mid/caudal part of the DRN at 1 d post-exposure (Figure 3). This was not seen in the rostral part (data not shown). The elevation in TPH2 mRNA levels was statistically significant in the KI model, albeit more clearly for the single (*p* < 0.0001) than for the double (*p* < 0.05) exposed group vs. their respective shams (Figure 3a). In the WRAIR model, a strong and significant increase in TPH2 transcript levels was observed after the second exposure (*p* < 0.0001, Figure 3a), whereas the increase after single exposure did not reach significance. Galanin mRNA was also elevated in this region in both the single (*p* < 0.01) and double (*p* < 0.01) exposure groups in the KI model (Figure 3b). Conversely, a significant increase was only found after the second exposure in the WRAIR model (*p* < 0.01, Figure 3b).

Importantly, we detected no cumulative effect of repeated blast exposure, i.e., single vs. double blast—neither for TH, TPH2, nor galanin mRNA levels, which were significantly higher in the double exposed groups of both model.

### Histopathology

Sections from the hippocampus were evaluated for degenerating neurons by FJ-B, and for axonal damage by staining for APP accumulation. We found no positive staining at this acute time point; hence, no evident cell degeneration or signs of axonal injury were detected in either exposed or sham rats in any of the groups (data not shown). Our previous study showed that there is no acute cell death or axonal pathology with the KI model (14). The immunohistochemisty was included to verify that the KI model behaves similar over time. For illustrations of cells that are positive to FJ-B and axons positive to APP the reader is referred to Risling et al. (14).

## Discussion

In this study, we directly compared the short-term effects of single and repeated mild injuries on the transcriptional response of genes involved in catecholamine, serotonin and galanin neurotransmission in the lower brainstem using two well-characterized models of bTBI. These models showed partial similarities with respect to single and repeated blast exposure relative to sham groups. But we found no cumulative effects after repeated exposure (i.e., single vs. double blast) under the experimental conditions used. Our findings highlight the importance of taking into account not only physical and technical parameters, but also environmental stressors when modeling mild bTBI. The importance of time, both in terms of inter-injury interval and post-injury termination, further emphasize the role of the tested transmitter systems in mediating responses in mild bTBI.

Modeling bTBI in the laboratory poses unique challenges due to the complexity of the physical forces resulting from explosive blast (12, 24, 25). Blast creates a highly complex injurious environment consisting of shockwaves and the blast wind, a high velocity (supersonic) air movement that can cause kinetic type of injury (26, 23). The closest to laboratory modeling of explosive blast affecting soldiers is using real explosives in a confined environment, as the blast tube used in the KI model (14, 23). This model, designed by Clemedson and Criborn (27). uses explosives resulting in high peak pressure and short positive phase duration (550 kPa and positive phase duration of 0.2 ms). The USU/WRAIR model is the more commonly used device that uses compressed air to generate the shock wave (12, 23). This model generates a lower peak pressure (average peak pressure 88.9 kPa) and a longer positive phase duration (8–9 ms) than the KI model. Whether the above differences in the physical parameters of blast generated by the two models can trigger different physiological responses is currently not known. However, the fact that the peak values of all analyzed transcripts were similar in all exposed animal groups, regardless of the blast model, suggest that both models trigger similar responses. This is true at least for our current outcome measures focusing on mRNA transcript levels for the rate limiting enzymes of noradrenergic and serotonergic neurotransmitters and galanin in the lower brainstem.

In contrast to the comparable injury-induced changes in transcript levels, sham values were significantly different in the KI and USU/WRAIR models. KI sham groups had significantly lower transcript levels than their corresponding USU/WRAIR sham groups. Statistically significant differences between exposed and sham animals of the KI model were likely due to relatively low sham values. Conversely, most of the values detected in the shams of the USU/WRAIR model were significantly higher than their respective KI shams. In fact, many sham values in the USU/WRAIR model were as high as exposed values seen in the KI model, implicating factors beyond physical blast parameters.

Among these factors are psychological stressors, which play a critical role in our selected neurotransmitter systems and can explain the detected differences in sham values. As detailed in the Methods, animals in the USU/WRAIR model were exposed to significant environmental stressors, associated with extended transportation, blast interleukin 6 levels in noises, and repeated handling. Consequently, in our earlier studies using the USU/WRAIR model we detected elevated corticosterone, interferon-γ, and interleukin 6 levels in sham animals relative to naïve animals not exposed to any stressors (28, 21). Using a battery of behavioral tests, the influence of different laboratory environments has also been demonstrated despite rigorous standardization between labs (29–31). Importantly, the transmitter systems we used as outcome measures have been known to be sensitive mediators of various stressors (32).

Intense activation of the noradrenergic system in the LC in response to stressful conditions has been clearly defined in the literature, and associated with adaptation to acute and chronic stress (33, 34). This in turn leads to activation of the noradrenergic terminals of the forebrain regions, including the cortex, with increases in noradrenaline (NA) turnover (35). A similar increase in TH mRNA levels in the LC was found following single or repeated (7x) daily immobilization stress (36). The neuropeptide galanin, which in rat co-exists with NA and 5-HT, in the LC and DRN (37), respectively, is also influenced by stress exposure (38). Chronic social stress studies revealed increased preprogalanin mRNA levels in the LC of subordinate rats that correlated with the number of wounds (39). Another study revealed an interesting effect on preprogalanin in two different strains of rats in the LC and central nucleus of the amygdala following acute and chronic restraint stress (40). With regard to the role of increased galanin in stress, it has been suggested that this peptide promotes stress resilience (41). An additional, important consideration when interpreting our results is that we only analyzed the transcriptional response at a single, acute time point. Our previous study has shown that the transcriptional response to mild blast using the KI model changes over time (17, 18).

The overwhelming majority of bTBIs are mild (42) and repeated (43). Clinical observations have shown that exposures to repeated mild blasts (as well as repeated concussions) can have cumulative effects resulting in more severe symptomatology after consecutive insults. Using the USU/WRAIR model, we have found evidence of a cumulative effect on brain microstructure using diffusion tensor imaging (DTI) (22). Diffusion tensor parameters had significant blast x no. of events (i.e., single vs. multiple) interactions in several subcortical brain regions including the stria terminalis, a key mediator of various autonomic and behavioral responses. Using the same model we also found significant behavioral and physiological changes after repeated mild blast exposure, including increased anxiety- and depression-related behaviors, elevated heart rate (up to 24 h post-injury), and increases in injury-related blood-based biomarkers (e.g., glial fibrillary acidic protein, neuron-specific enolase, and neurofilament) (44, 45). These and other studies (46) have demonstrated the critical role of inter-injury interval in causing cumulative damage.

The exact period of increased cerebral vulnerability after injury is currently not unknown, but it can be as long as several weeks in humans. Rats have a very different physiology compared to humans, and experiments have shown that such a window can be as short as 30–60 min in rats (28, 22, 47, 48). However, these and other experiments have indicated that metabolic, inflammatory, axonal, and vascular functions (among other processes) have differing periods of increased cerebral vulnerability after injury. Importantly, and of particular relevance for this study, isoflurane post-conditioning has been explored as a possible therapeutic, as it can reduce brain infarction size and attenuate neurological deficits (49). Given that our blast-exposed animals were repeatedly anesthetized with isoflurane, this could account for the absence of a cumulative effect and/or the mitigation of subsequent injury mechanisms in the double blast groups.

## Limitations and concluding remarks

There is an ongoing and important discussion concerning a link between long-term neurodegenerative pathologies, such as chronic traumatic encephalopathy (CTE). and repeated mild TBI. This concern includes both blast exposure and occupational aspects of the use of heavy weapons. While the majority of CTE cases have been reported in contact sports like American football or Ice hockey. there are a few cases among military veterans with a history of blast exposure (50). There are also a few interesting reports in tau related pathology in mice models of concussion or blast exposure (51, 52). The current study was not designed to reveal specific information on possible neurodegenerative changes at later time points, so we cannot make statements on a possible relation to CTE. However, some studies suggest a link between the function of the monoaminergic systems and the progression of dementia (53).

This is the first study to our knowledge that has directly compared two well characterized and frequently used experimental rodent models of blast, examining changes in select neurotransmitter and peptide systems following single and repeated exposures. The limitations of our study include the relatively low number of subjects used in the different experimental groups, the single post-injury time point of sampling for analysis, and the single, short inter-injury interval separating repeated exposures. Also, we have only investigated blast effects at the mRNA levels of TH, TPH2 and galanin. How these transcriptional changes translate into changes at the protein level remains to be studied.

There have been significant efforts to elucidate the neurological underpinnings of mbTBI and its long-term sequelae. The monoamine systems have since long been associated with mood disorders (54), and recent studies indicate that this may also be true for galanin (55). However, translation between rodent models and clinical cases is difficult. Our findings highlight the critical importance of mimicking not only the physical parameters of blast, but also the environmental factors. They can, as our data show, majorly affect post-injury outcomes. Given its blast profile and optimal animal handling conditions, the KI model offers a useful experimental system for determining the period of increased cerebral vulnerability, and dissecting the effects of physical blast forces on this and other transmitter systems, from bTBI and concomitant psychological insults.

## Disclosure

The views, opinions, and/or findings contained herein are those of the authors and should not be construed as an official position, policy, or decision of the Department of the Army or the Department of Defense.

## Author contributions

LK carried out all tissue preparation at KI and all *in situ* hybridization and immunohistochemistry experiments. LK was also responsible for all the data analysis and was involved in planning and writing. AK carried out blast experiments and tissue preparation at USU. AK was also involved in involved with planning, and writing. JL associations and use of blast facilities at WRAIR. UA carried out blast experiments at KI. TH senior professor involved with planning, writing, and interpreting data. DA senior author at USU and KI, involved with planning, writing, and interpreting data. MR senior author at KI, involved with planning, writing, and interpreting data.

### Conflict of interest statement

The authors declare that the research was conducted in the absence of any commercial or financial relationships that could be construed as a potential conflict of interest.

## Abbreviations

bTBI: blast-induced traumatic brain injury
mbTBI, mild blast-induced traumatic brain injury, ISH: *in situ* hybridization
LC: locus coeruleus
PTSD: post-traumatic stress disorder
DRN: dorsal raphe nucleus.
